# Reactivation of latent HIV-1 *in vitro* using an ethanolic extract from *Euphorbia umbellata* (Euphorbiaceae) latex

**DOI:** 10.1371/journal.pone.0207664

**Published:** 2018-11-27

**Authors:** Ana Luiza Chaves Valadão, Paula Pezzuto, Viviane A. Oliveira Silva, Barbara Simonson Gonçalves, Átila Duque Rossi, Rodrigo Delvecchio da Cunha, Antonio Carlos Siani, João Batista de Freitas Tostes, Marcelo Trovó, Paulo Damasco, Gabriel Gonçalves, Rui Manuel Reis, Renato Santana Aguiar, Cleonice Alves de Melo Bento, Amilcar Tanuri

**Affiliations:** 1 Laboratório de Virologia Molecular, Departamento de Genética, Universidade Federal do Rio de Janeiro, Rio de Janeiro, Brazil; 2 Centro de Pesquisa em Oncologia Molecular, Hospital de Câncer de Barretos, Barretos, Brazil; 3 Departamento de Microbiologia e Parasitologia, Universidade Federal do Estado do Rio de Janeiro, Rio de Janeiro, Brazil; 4 Departamento de Produtos Naturais, Instituto de Tecnologia em Fármacos, Fundação Oswaldo Cruz, Rio de Janeiro Brazil; 5 Departamento de Botânica, Instituto de Biologia, Universidade Federal do Rio de Janeiro, Rio de Janeiro, Brazil; 6 Life and Health Sciences Research Institute (ICVS), School of Medicine, University of Minho, Braga, Portugal; 7 ICVS/3B’s–PT Government Associate Laboratory, Braga/Guimarães, Portugal; Cleveland Clinic, UNITED STATES

## Abstract

*Euphorbia umbellata (E*. *umbellata)* belongs to *Euphorbiaceae* family, popularly known as Janauba, and its latex contains a combination of phorbol esters with biological activities described to different cellular protein kinase C (PKC) isoforms. Here, we identified deoxi-phorbol esters present in *E*. *umbellata* latex alcoholic extract that are able to increase HIV transcription and reactivate virus from latency models. This activity is probably mediated by NF-kB activation followed by nuclear translocation and binding to the HIV LTR promoter. In addition, *E*. *umbellata* latex extract induced the production of pro inflammatory cytokines *in vitro* in human PBMC cultures. This latex extract also activates latent virus in human PBMCs isolated from HIV positive patients as well as latent SIV in non-human primate primary CD4^+^ T lymphocytes. Together, these results indicate that the phorbol esters present in *E*. *umbellata* latex are promising candidate compounds for future clinical trials for *shock and kill* therapies to promote HIV cure and eradication.

## Introduction

The Acquired Immunodeficiency Syndrome (AIDS) is caused by the Human Immunodeficiency Virus (HIV), which was identified in 1983 [1,2]. Since then, more than 35 million people have died from AIDS-related illnesses all over the world [3].

The pandemic of HIV infection is an important socioeconomic burden and is considered one of the largest documented epidemics in history. HIV pathogenesis starts with the infection and replication of the virus in CD4^+^ T lymphocytes, macrophages and dendritic cells. Replication and destruction of CD4^+^ T cells, which are key effectors of the host immune response, leads to the clinical outcome of immunosuppression known as AIDS [4].

The current strategy used as a therapy for HIV infection is named Antiretroviral Therapy (ART) and is based on a combination of antiretroviral drugs that target different stages of HIV replicative cycle. The primary goal of ART is to block viral replication and consequently slow down or even stop the progression to AIDS, restoring immunity and improving the quality of life of the infected patient [5]. However, adverse effects have been reported as a consequence of ART in conjunction with immunological, neurological and metabolic co-morbidities associated either with HIV infection or drug interactions [6]. These facts triggered the search for a functional or sterilizing cure for HIV infection.

After achieving an undetectable viral load with effective antiretroviral therapy, however, it is still possible to detect latent viral reservoirs where HIV proviral DNA is integrated in resting memory CD4+ T cells [7]. These cells containing latent viral genomes become viral reservoirs that are inaccessible to ART. Then, if ART is stopped, viral load increases rapidly and systemic infection is reestablished [8]. Therefore, the complete eradication of HIV from an infected individual is one of the most challenging areas of HIV research today. In order to eliminate HIV it’s necessary to activate viral reservoirs in the presence of ART or stimulate the immune response to destroy them.

New latent virus reactivating compounds, also called latency reversing agents (LRA), when used in combination with ART, are able to eliminate transcriptionally inactive viruses, ultimately resulting in the eradication of HIV infection. Histone deacetylase (HDAC) inhibitors are a promising class of latency-reversing agents (LRAs) that are undergoing extensive *in vitro* testing and clinical trials to reactivate latent HIV-1 infections. HDAC inhibitors were developed as anticancer drugs due to HDACs important role in epigenetic and non-epigenetic transcriptional regulation, inducing apoptosis and cell cycle arrest [9]. In the context of HIV-1 reactivation, HDAC inhibitors promote transcription of the HIV-1 long terminal repeat (LTR) [10–13]. This strategy is called “shock and kill” and it postulates that memory CD4^+^ T lymphocytes treated with LRA along with ART could purge totally or partially the viral reservoirs while blocking replication of emergent latent virus, leading to a HIV eradication or at least a functional cure [14]. Another promising class of molecules with therapeutic potential as possible candidates for reactivation of viral reservoirs are the Protein Kinase C (PKC) agonists [7]. This class includes Prostratin [15], Bryostatin [16], Ingenol [17] and Phorbol 12-Myristate 13-Acetate (PMA) [18]. All these compounds can reactivate latent HIV in lymphoid and myeloid cells.

Although several transcriptional regulatory mechanisms for HIV latency have been described in the context of epigenetic silencing and transcription repression, recent work suggests that reactivation of latent HIV is positively correlated with NF-κB activation, suggesting the importance of this factor to latency reactivation [19,20]. Many natural compounds have been investigated for their antiviral and latency-reversing properties, being indicated as candidates for clinical trials. Several of these compounds are derived from secondary metabolics of plants, such as terpenoids [21], polyphenols [22], alkaloids [23] and phorbol esters [24]. The *Euphorbiaceae* family is a known source of potent phorbol-esters, with several specimens described worldwide, and has been used for the treatment of diseases in the popular medicinal use [25–29]. *Euphorbia umbellata* is popularly known in Brazil as Janauba or *leiterinha* and is used, in traditional medicine, as a latex preparation (diluted in water) for the treatment of gastric disorders, such as peptic ulcer and gastritis, as well as neoplastic diseases, allergy, diabetes, influenza, leprosy, among other diseases [30]. Several papers described isolation of lectins, phorbols, terpenes, flavonoids and other phenolic compounds from *E*. *umbellata* latex [31,32]. This plant contains phorbol esters in its latex that are similar to PMA, which is isolated from the seeds of *Croton tiglium*, another representative of the *Euphorbiaceae* family. These compounds are efficient at inhibiting the cytopathic effect generated by HIV infection on lymphocyte cells, as well as activating protein kinase C (PKC) that in turn promotes NF-κB translocation into the nucleus, activating HIV-1 from latency [33].

In this work we evaluated the ability of an ethanolic extract obtained from Janauba latex (JALEx) to reactivate latent HIV-1 and investigated the molecular mechanisms involved in this activity. The anti-latency property of JALEx was demonstrated either *ex vivo* in HIV latent derived human PBMC cells or in CD4+ T Lymphocytes isolated from SIV infected non-human-primates. All these properties make JALEx a promising natural medicine to be used in HIV cure strategies.

## Material and methods

### Latex isolation from *Euphorbia umbellata*

*Euphorbia umbellata* (Pax) Bruyns, the current accepted name for *Synadenium grantii* Hook.f., was collected and identified with the respective voucher specimen kept at the RFA Herbarium under the number Trovó, M.L.O 764. The crude latex was collected directly from an incision mechanically made in the trunk of cultivated specimens at Barretos city, São Paulo state, Brazil. The latex was straightly diluted in 80% ethanol (1:10 v/v) to produce a white rubber-like precipitate that was filtered off by gravity using filter papers. The filtrate was dried in a rotary evaporator to reach a fine white powder that was diluted in DMSO to a final concentration of 20 mg/mL. This extracted material was labeled as JALEx (Janaúba Alcoholic Latex Extract). JALEx was chromatographed in a silica gel column using a solvent gradient (0–5%) of ethyl ether in chloroform to the enrichment of phorbol-containing fractions, allowing its detection and characterization.

### Chemical profile of JALEx

JALEx was analyzed by gas chromatography combined with mass spectrometry (GC-MS) using an Agilent 6890N chromatograph (Palo Alto, CA) equipped with a capillary column DB-H17HT (30 m × 0.25 mm × 0.15 μm film thickness). Helium was the carrier gas at 1.0 mL/min and inlet pressure of 3.14 psi, 10:1 split/splitless ratio. The oven temperature was programmed from 150°C to 340°C at 4°C/min for 17 minutes. The injection volume was 1 μL of a 2 mg/mL chloroform solution. Data were processed using MSD Productivity Chem Station Software operating with ion source at 250°C and electron impact ionization at 70 eV. Individual components were characterized by their fragmentation patterns in the mass spectrum.

### Viruses and cells

To analyze HIV-1 reactivation in latent infected cells after treatment with JALEx, Human HIV-1 latent lymphocyte cell models (J-Lat 8.4 and 10.6) were used [34]. These cells are derived from Jurkat cells containing copies of HIV-1 genome in fusion with GFP reporter gene integrated into the nuclear [34]. These cells differ on their response to reactivation due to differences on their site of HIV-1 integration. The J-Lat 10.6 clone is more responsive, showing higher levels of reactivation than those observed for the J-Lat 8.4 clone. Both cells were cultured in RPMI medium supplemented with 10% fetal bovine serum (FBS) and 1% penicillin / streptomycin antibiotics. An amount of 10^5^ J-Lat cells per well of 24-well plates were treated for 24 hours with different JALEx concentrations using DMSO as diluent. As positive controls for reactivation of HIV-1 latent virus were used ingenol B (0.32 μM) and PMA (1 μM). HIV-1 transcription/reactivation was monitored by GFP detection with flow cytometry (BD Accuri C6) assays. A total of 10,000 gated live cells were collected and data is presented by the percentage of GFP-expressing cells in total gated events. All the experiments were performed in three independent replicates.

### Cell viability assay

The cytotoxicity of the compounds was assessed by incubating J-Lat cells with serial dilutions of JALEx extracts previously tested in the reactivation assays. Cell viability estimation was performed in 10^4^ cells plated on 96-well plates in three independent experiments, each one with six technical replicates. After 5 days of cell culture, vital resazurin dye (CellTiter Blue, Promega) was added to the cells and absorbance levels were evaluated by spectrophotometer at 595 nm.

### Analysis of the activation of PKCs

To investigate the mechanism of action of HIV-1 latency reactivation by Janauba compounds, 10^5^ J-Lat cells were plated on 24-well plates were treated with different specific inhibitors of PKCα, PKCδ and PKCγ isoforms Go6976, Go6983, Ro-31-8220, respectively, for 24 hours [35]. After this time, cells were treated with a positive control of PKC activation (1 μM PMA) and two concentrations of JALEx previously determined in the reactivation and cytotoxicity assays. Cells treated only with the diluent (DMSO) were referred as MOCK. The following concentrations of the alcoholic extract were tested: 0.01 μg/mL and 0.001 μg/mL.

In addition, PKC activation was also evaluated by immunofluorescence to detect different PKC isoforms. For this, 10^4^ HeLa cells were plated on black 96-well glass bottom plates. These cells were transfected with three different plasmids encoding three different PKC isoforms fused to GFP (PKCα-GFP, PKCδ-GFP and PKCγ-GFP) for 24 hours and then treated with 1 μg/mL and 0.1 μg/mL of the JALEx, for 10 minutes or 24 hours. Positive control was performed using PMA (1 μM). Cells were then washed with PBS and the nucleus was evidenced by DAPI staining. Cellular localization of PKC-GFP was asssessed by High Content Screening confocal microscope (Molecular Devices, Inc). The redistribution of PKC isoforms was quantified in ImageJ software.

Phosphorylation of PKC isoforms was also investigated. For this, Jurkat cells were treated with 0.01 μg/mL and 0.001 μg/mL of JALEx and with 1 μM PMA as positive control for 10 and 30 minutes, 1, 6 and 24 hours. Cellular extracts were obtained by incubation with lysis buffer (50 mM Tris pH 7.6, 150 mM NaCl, 5 mM EDTA, 1 mM Na_3_VO_4_, 10 mM NaF, 10 mM NaPyrophosphate, 1% NP-40 supplemented with protease inhibitors cocktail) for 1 hour. Lysates were then subjected to 10% SDS-PAGE gels and after transferred to nitrocellulose membranes the following antibodies were used for immunoblotting: anti-PKC phosphorylated PKC (pan, δ, θ) (Cell Signaling; 1:500) and β-tubulin (Cell Signaling; 1:2000). Proteins quantification was evaluated using band densitometry analysis, performed with the Image J software (version 1.41; National Institutes of Health).

### NF-κB activation assays

In order to verify whether JALEx induces the activation and downstream nuclear internalization of NF-κB, HeLa cells were plated at the density of 2x10^4^ cells per well in 96-well plates. Cells were treated with 1 μg/mL and 0.1 μg/mL of JALEx for 6 or 24 hours. PMA (1 μM) was used as positive control. The NF-κB subunit p65 was visualized by immunofluorescence (anti-p65, 1:300, Santa Cruz Biotechnologies), and nuclear (DAPI) translocation was evaluated by fluorescence confocal microscopy (Molecular Devices, Inc).

To investigate the possible involvement of NF-κB in reactivation of latent HIV-1, Jurkat cells (10^6^ cells) were transiently transfected with 8 μg of pBlue30LTR-Luc Wild Type (NFkB WT) or p-LTR-MUT (NFkB MUT) using the Neon Transfection System kit (Thermo Fisher Scientific) according to the manufacturer's recommendations. The latter plasmid was generated through site-directed mutagenesis by altering the two NF-κB binding sites, making that plasmid nonfunctional for NF-κB response. Both plasmids have the luciferase sequence under the control of the HIV-1 viral promoter. In the case of plasmid carrying NF-kB mutated sequence, the second guanine at both NF-κB binding sites into LTR promoter was changed to a cytosine, resulting in the plasmid designed here as *pBlue3*′*LTR NF-κB MUT-Luc* (NF-κB BS^−^). This plasmid carries a LTR sequence lacking functional NF-κB binding sites as described before [17]. After transfections, cells were equally divided into two groups: treated or untreated with 1μg/mL of JALEx, for 24 hours. After this interval, cells were lysed and luciferase activity was measured with a luminometer (Glomax, Promega).

### Analysis of surface markers expression and cytokines production in primary cells treated with JALEx

In order to expand the results obtained in HIV-1 latency model cells, we performed JALEx treatment assays with peripheral blood mononuclear cells (PBMCs) from 4 healthy donors. After buffy coat separation by Ficoll centrifugation, CD4^+^ T cells were isolated using the Dynabeads CD4 Positive Isolation Kit (Thermo Fisher) kit to obtain 10^7^ PBMCs, according to the manufacturer's recommendations. TCD4^+^ cells were treated with 1 μM PMA, 10 μg/mL (10^−3^) or 1 μg/mL (10^−4^) of JALEx, in addition to MOCK (DMSO-treated cells), for 24 hours. Expression of surface markers such as CD4, CCR5 and CXCR4, as well as the expression of activation markers CD25, CD38, HLA-DR and CD69 were evaluated by specific antibody labeling and flow cytometric analysis (BD Accuri C6). Comparatively, this assay was also performed on MT4 lymphocyte cells line at the same conditions.

Cell supernatants from the CD4+ T cells were collected for analysis of the cytokine production stimulated by JALEx treatment. A multiplex immunoassay Bio-Plex Pro Human Cytokine 17-plex Assay (Bio-Rad) containing fluorescent microspheres conjugated to different monoclonal antibodies specific for each target cytokine was chosen for this analysis. The following cytokines were measured: IL-1β, IL-2, IL-4, IL-5, IL-6, IL-7, IL-8, IL-10, IL- IL-17, G-CSF, GM-CSF, MCP-1 / MCAF, MIP-1β, IFNγ and TNF-α.

### Ethic statement

The use of primary human cells in the present study was approved by the Institutional Review Board at Universidade Federal do Estado do Rio de Janeiro under approval number 30286514.4.0000.5258. Written consent was obtained from all participants.

### Quantitative viral outgrowth assay

To compare the ability of the compounds to stimulate virus replication in latently infected cells we used a variation of the quantitative viral outgrowth assay (QVOA) described by Laird et al. [36]. For the QVOA, cells were isolated from viremic SIVmac239-infected cynomolgus macaques at the Wisconsin National Primate Research Center (WNPRC). PBMCs obtained by density gradient centrifugation were enriched for CD4^+^ T cells using the CD4 T cell enrichment kit for non-human primates (Miltenyi Biotec, San Diego, CA). After enrichment, cells were plated at 1x10^6^ cells per well. These cells were co-cultured with “stimulator” CEMx174 cells that were irradiated with 10,500 rads prior to co-culture. The ratio of stimulator cells to CD4^+^ T cells was 2:1. Purified CD4^+^ T cells were stimulated with JALEx, concanavalin A, ingenol, or no mitogen, overnight. After stimulation, cells were washed and re-plated in serial dilutions, in triplicate. At this point, additional non-irradiated CEMx174 cells were added to each well to serve as targets for induced virus to infect. The target cells were added at a 2:1 ratio to the CD4^+^ T cells. Co-cultures were maintained for 1 week. On the last day, supernatant was collected and RNA was extracted to test for the presence of SIV. RNA extraction was made by the Viral Total Nucleic Acid kit for the Maxwell 16 MDx instrument (Promega, Madison, WI). Viral RNA was quantified using a qRT-PCR assay targeting the *gag* gene as described previously [37].

### Effect of JALEx in T cell activation from HIV-1 positive patients

In order to analyze the ability of JALEx to regulate different T cell phenotypes in the context of HIV-1 infection, samples of the peripheral blood of 15 adults ART-treated patients (8 women and 9 men), with a mean plasma viral load of 953 ± 1.924 copies of RNA/mL and CD4^+^ (845 ± 345/mm^3^) and CD8^+^ (845 ± 345/mm^3^) higher than 350 cells/mm^3^, were collected and PBMC were obtained from Ficoll-Paque gradient. Viable PBMC were quantified by trypan blue exclusion and suspended in RPMI-1640 medium supplemented with 2 μM of L-glutamine (GIBCO, Carlsbad, CA, USA), 10% of fetal calf serum, 20 U/mL of penicillin, 20 μg/mL of streptomycin and 20 mM of HEPES buffer. Approximately 1 x 10^6^ PBMC/mL were cultured for 24 hours on 24-well plates with 1mL of complete medium in the presence or absence of different concentration of JALEx (1, 0.1 and 0.01 μg/mL). Phorbol-12-myristate-13-acetate (PMA 20 ng/mL) plus ionomycin (600 ng/mL) were used as the positive control. To optimize intracellular cytokine staining, brefeldin A (10 μg/mL) was added to the cultures in the last 4 hours of incubation time. For the fluorescence labeling, mouse anti-human fluorescent monoclonal antibodies (mAbs) directed against CD4-FITC, CD8-FITC were purchased from BD Bioscience (San Diego, CA, USA). Briefly, anti-CD4 and anti-CD8 mAbs were added to the PBMC (2 x 10^5^/tube) and incubated for 30 min at room temperature in the dark. Cells were washed with PBS and then permeabilized with Cytofix/Cytoperm (BD Pharmingen, San Diego, CA) at 4°C for 20 min. After washing, antibodies for intracellular staining (anti- IL-17-PECy7, anti-IFN-γ-PE-Cy5, anti-IL-10-PE-Cy7, anti-IL-21-PE) or the corresponding anti-IgG1 isotype control were added in various combinations and incubated for 30 min at 4°C. Cells were analyzed in the Attune (Thermo Fischer, Co) and FlowJo software. Isotype control antibodies and single-stained samples were periodically used to check the settings and gates at the flow cytometer. After acquisition of 100,000 to 200,000 events, lymphocytes were gated based on the forward and side scatter properties after the exclusion of dead cells, by using propidium iodide and doublets. The viability of CD4^+^ and CD8^+^ cells was determined using 7-aminoactinomycin D (7-AAD) (R&D systems). Further, gated cells were negative for CD14 marker. The CD4^+^ T lymphocyte count (CD4) was performed on a flow cytometer (FACS Count, Becton Dickson). Plasmatic HIV-1 RNA quantification was performed using the RT-PCR with Abbott M2000 Sq HIV VL technology.

### Statistical analysis

The statistical analysis was performed using Prism 5.0 software (GraphPad Software). Statistical evaluations were estimated using Kruskal-Wallis Test, Mann–Whitney U test and Two-way ANOVA. Tests are indicated in figure legends.

## Results

### JALEx isolation from *Euphorbia umbellate and chemical analysis*

**Fig 1:**
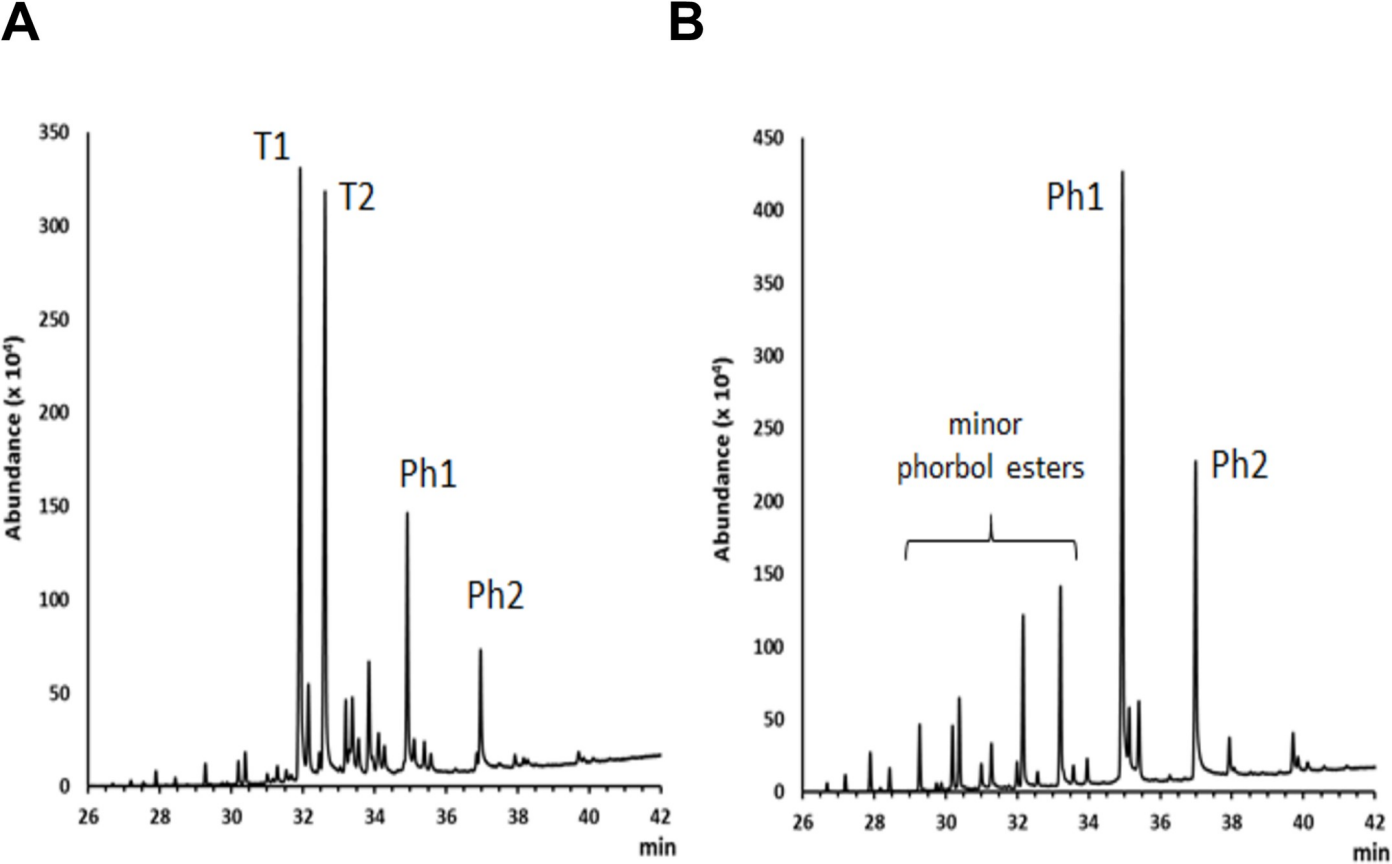
Isolation of JALEx. Gas chromatograms of (A) whole extract JALEx and (B) Phorbol ester-enriched JALEx fractions. T1 and T2 = major triterpenes constituents; Ph1 and Ph2 = major phorbol esters constituents. The chemical classes were indicated by the characteristic mass fragmentation in the GC-MS analysis and data from literature.

JALEx is soluble in water and ethanol and was characterized mainly by a mixture of triterpenes and phorbol ester diterpenes. The GC profile and MS data indicated that triterpenes were predominant throughout the whole extract, with two main constituents standing out, represented as T1 and T2 in Fig 1A. The triterpenes profile is in accordance with the data from literature that report the isolation of euphol, lanosterol, friedelin and 3β-friedelinol from the latex, leaves and stem of *E*. *umbellata* [38], whereas studies with the latex have led to the characterization of euphol and the steroid citrostadienol [39]. A phorbol ester-rich fraction was obtained by chromatographing crude JALEx in a silica gel open column using mixtures of chloroform-ether as eluent (Ph1 and Ph 2—Fig 1). The class of phorbol ester has been represented by the 12-O-tigloyl-4-deoxyphorbol-13-isobutirate [40], 12-deoxyphorbol-13-(2-methylpropionate), phorbol 12,13,20-triacetate [41], 4-deoxyphorbol-12,13-ditiglate and 3,4,12,13-tetraacetylphorbol-20-phenylacetate [42] distributed in several plant tissues (S1 Fig).

### Reactivation of latent HIV-1 by JALEx and its cytotoxicity effects

**Fig 2:**
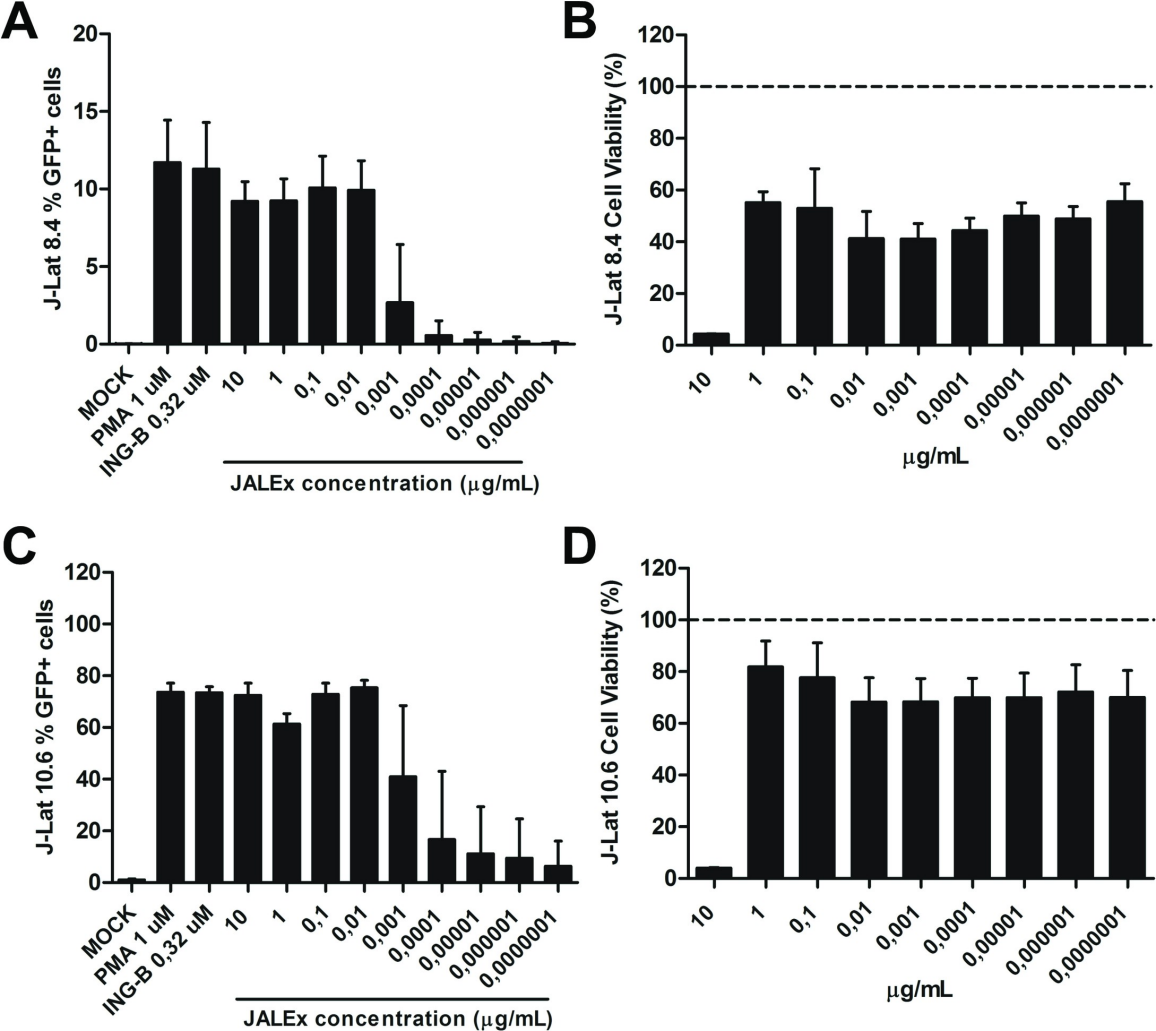
JALEx reactivates virus transcription in HIV-1 latent models. J-Lat 8.4 (A) and 10.6 (C) cells were exposed to increasing concentrations of JALEx and HIV-1 activation was assessed 24 hours post-treatment by GFP positive cells screening with flow cytometry assay. Mock stands for DMSO treated cells. PMA (1 μM) and INGB (0.32 μM) were used as positive induction controls. The cytotoxicity of JALEx was evaluated using CellTiter Blue vital stain 5 days after treatment in J-Lat 8.4 (B) and 10.6 cells (D). GFP positivity and cytotoxicity were expressed as a percentage of cells relative to mock (DMSO-treated) cells (n = 3). Dashed lines in Fig 2B and 2D represents 100% of viability measures in MOCK. The bars in the Fig represent the SD around the mean.

In this work, we used ethanolic extracts from JALEx that were dried and then resuspended in DMSO. The reactivation of latent HIV-1 was evaluated using the lymphocytic lineage J-Lat cells (clones 8.4 and 10.6) harboring the latent HIV-1 integrated genome. Cells were exposed to increasing concentrations of JALEx, ranging from 10 to 10^−7^ μg/mL. The reactivation of latent HIV-1 provirus was evaluated by expression of GFP reporter gene cloned into the virus-integrated genome. In parallel, cytotoxicity assay was performed to evaluate whether concentrations of JALEx would be toxic to these cells. JALEx treatment at concentrations of 10 to 0.01 μg/mL showed HIV-1 activation percentage similar to positive controls (PMA 1 μM and ING-B 0.32 μM), dropping to less than 5% reactivation at dilutions of 0.001 to 0.0000001 μg/mL in the J-Lat 8.4 clone (Fig 2A). Our results showed cytotoxic effects of JALEx at 10 μg/mL, while other concentrations were well tolerated, showing no significant changes in cell viability (Fig 2B). As expected, HIV-1 reactivation by JALEx was more expressive in J-Lat 10.6 cells, reaching 75% of GFP-positive cells while only 12% of 8.4 cells showed reactivation. (Fig 2C). In relation to the cytotoxicity, JALEx at 10 μg/mL was also toxic for J-Lat 10.6, whereas other concentrations presented cell viability between 80% and 75% (Fig 2D). These results demonstrated the ability of JALEx to stimulate HIV-1 transcription and to reactivate virus from latency models.

### JALEx activation of different isoforms of PKC

Preliminary data using PKC inhibitors Gö6666, Gö6983 and Rö31-8220 suggest that JALEx reactivation of latent HIV-1 was mediated by its ability to activate PKC (S2 Fig). Therefore, we investigated its effect on PKC activation and cellular translocation.

PKC isoforms are divided into three subgroups: the conventional isoforms (α, βI, βII and γ) that are Ca^2+^and DAG dependent for activation; the novel class encompassing (δ, ε and η) that require DAG, but not Ca^2+^ for activation and the atypical isoforms (ι and λ) that requires neither Ca^2+^ nor diacylglycerol for activation. For these reasons, we investigated whether phosphorylation of different PKC isoforms was occurring in response to JALEx treatment. In these experiments, two concentrations of JALEx (0.01 μg/mL and 0.001 μg/mL) were used and PMA was utilized as the positive control of PKC phosphorylation. Cells were collected at different time points (10 and 30 minutes; 1, 6 and 24 hours). We observed that the phosphorylation of conventional PKCs occurs quite rapidly, after 10 minutes of treatment with JALEx, in both concentrations tested (S3 Fig). Moreover, this activation is long lasting, remaining phosphorylated for up to 24 hours after the treatments (S3 Fig). The same pattern was observed at the same time points for cells treated with PMA (S3 Fig). About the novel PKC isoforms, PKC θ exhibited a rapid phosphorylation pattern (10 min) that was not sustained over time (1 to 24h) (S3 Fig). PKC δ showed an early phosphorylation pattern maintained for up to 6 hours with JALEx treatment in both concentrations (0.01 μg/mL and 0.001 μg/mL). However, only JALEx at 0.001 μg/mL induces PKC δ phosphorylation after 24 hours (S3 Fig). Densitometry analyses compared with loading controls (α-tubulin) and normalized by DMSO treated cells showed that, in general, JALEx at concentrations of 0.01 μg/mL and 0.001 μg/mL induce the phosphorylation of conventional and novel isoforms of PKC that remains at least up to 6 hours of stimulation (S3 Fig). However, as suggested by the western blot analysis, the phosphorylation of PKC-δ is sustained until 24 hours post JALEx treatment. All these results are in agreement with the previous observations that JALEx is a pan activator of PKC isoforms.

**Fig 3:**
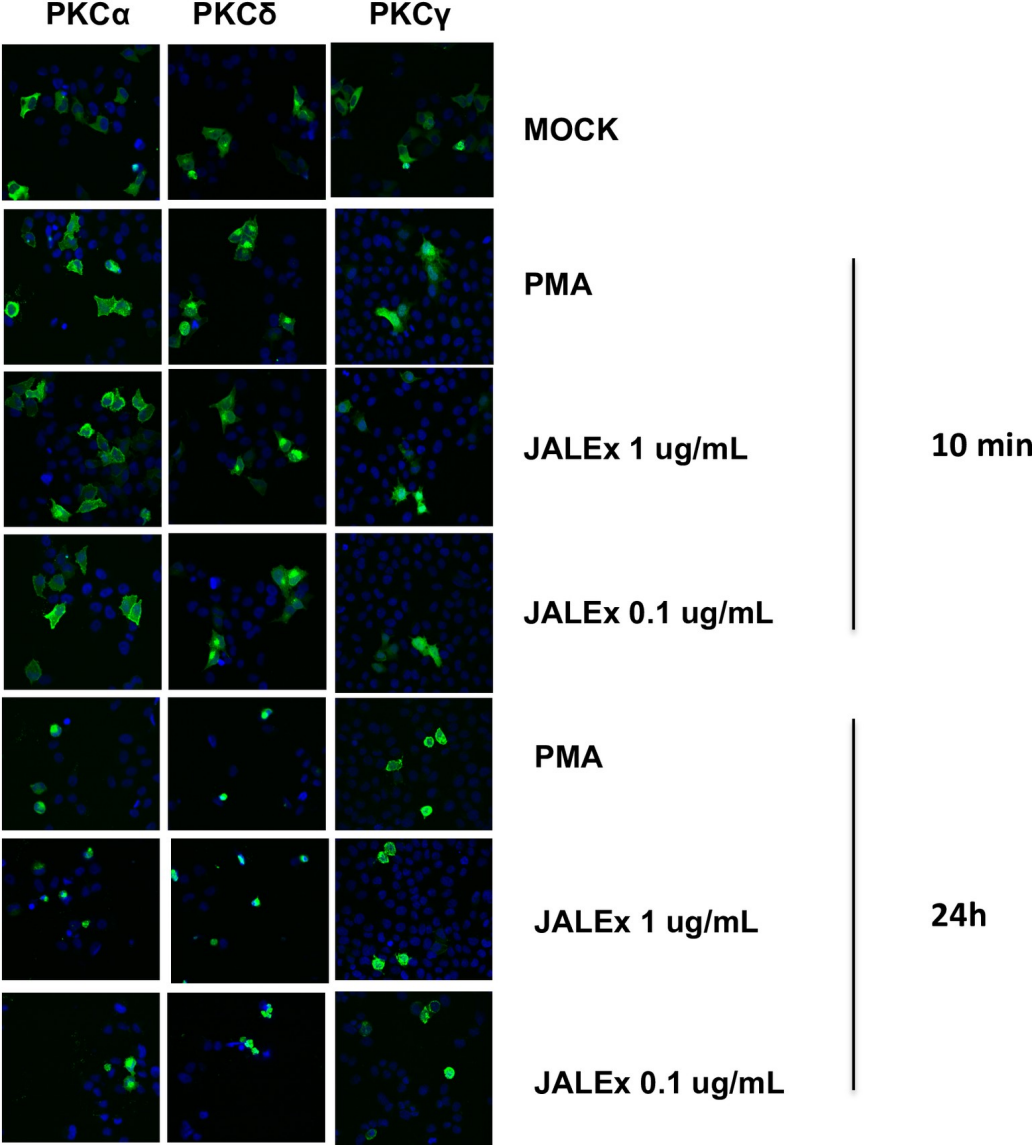
JALEx induces cellular relocation of PKC isoforms. Cellular location analysis of different PKC (alpha, delta and gamma) isoforms after treatment of HeLa cells with two concentrations of JALEx: 1 μg / mL and 0.1 μg / mL. All cells were submitted to nuclear staining with DAPI (blue). HeLa cells were transfected with 3 different PKC isoforms for 24h and treated with JALEx for 10 min and 24 hours. PMA (1 μM) was used as positive control (n = 3) and Mock images were taken with the results from 24 hours.

After activation, PKCs are translocated to the plasma membrane by RACK proteins (membrane-bound receptor for activated protein kinase C proteins) [43]. To verify if JALEx promotes the translocation of PKC isoforms to the plasma membrane, HeLa cells were transfected with plasmids expressing three PKC isoforms fused to GFP (PKCα-GFP, PKCδ-GFP and PKCγ-GFP). After transfection, cells were treated with 1 and 0.1 μg/mL of JALEx for 10 minutes or 24 hours and the cytoplasmic location of PKC were evidenced by fluorescence confocal microscopy images. We observed a diffuse pattern of PKC distribution in DMSO-treated cells (mock). However, PMA or JALEx treatment at both concentrations (1 and 0.1 μg/mL) induced the periplasmatic distribution of all PKC isoforms (α, δ and γ) with longer a treatment scheme (up to 24h) (Fig 3). These results show that JALEx promotes activation of different classes of PKC through their phosphorylation followed by their downstream relocation at the plasma membrane.

### JALEx promotes nuclear translocation of NF-κB and requires an intact NFkB binding site on the HIV-1 promoter to induce reactivation

**Fig 4:**
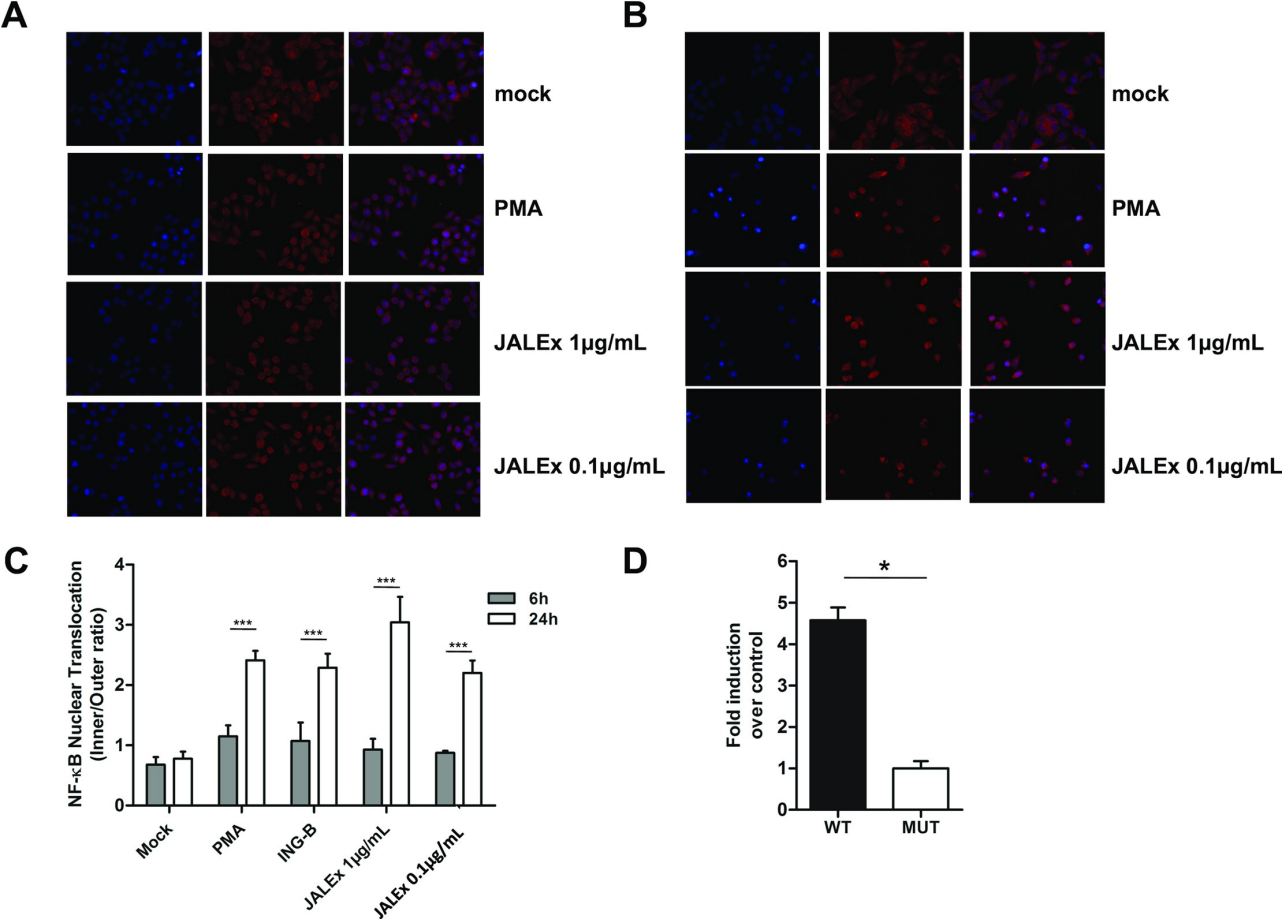
JALEx-mediated HIV-1 transcriptional activation by NF-κB nuclear translocation. HeLa cells were treated with two concentrations of the JALEx (1 and 0.1 μg/mL) for 6 hours (A) and 24 hours (B). After, cells were subjected to immunofluorescence labeling of NF-κB (red) and cell nucleus (blue). As a positive control, PMA (1 μM) was used. (C) Quantification of NF-κB nuclear translocation of 100 HeLa cells for each condition (n = 3; Two-way ANOVA and (***) indicate p<0,0001). (D) Jurkat cells were transfected with pBlue30LTR-Luc (NF-κB WT) and the mutant construct, pBlue30LTR NF-κB MUT-Luc (NF-κB MUT), which is absent for the NF-κB binding site. After transfections, cells were treated with 1μg / mL of the JALEx for 24 hours and luciferase expression was measured after lysis. The fold-change of the luciferase expression between the wild-type plasmid and the mutant is shown in the graph (n = 4, Mann-Whitney test and (*) indicate p<0,05). The bars in Fig 4C and 4D represents the SD around the mean.

Phosphorylation of NF-kB inhibitor IκB by PKC leads to its ubiquitination and proteasomal degradation, releasing NF-κB complexes in the cytoplasm to translocate to the nucleus where they bind to HIV-1 promoter (LTR region) and induce virus gene expression [15,44,45]. For this reason, we evaluated the effect of JALEx treatment on nuclear translocation of NF-κB two different strategies. In the first one, we analyzed the occurrence of nuclear translocation of NF-κB after treatment of HeLa cells with 1 and 0.1 μg/mL of JALEx for 6 or 24 hours, through immunofluorescence and confocal fluorescence microscopy using anti-p55 antibody. We observed no significant translocation of NF-κB to the nucleus of HeLa cells 6 hours post JALEx treatment with the same cytoplasmic distribution as observed in untreated cells (mock—Fig 4A). However, we confirmed that NF-kB is translocated to the nucleus after 24 hours of JALEx treatment (Fig 4B). To better quantify this translocation of NF-xB, we quantified the mean values of fluorescence intensity between the nucleus and the cytoplasm of 100 cells, in each experimental condition. We observed an increasing ratio of NF-kB fluorescence into the nucleus 24 hours after JALEx treatment, confirmed by other NF-kB activators such as PMA and ING-B (Fig 4C). JALEx treatment at the concentration of 1 μg/mL showed the highest rates of NF-kB internalization (Fig 4C).

We also evaluated the involvement of NF-κB translocation on HIV promoter activation by JALEx treatment. For that, we transfected Jurkat T CD4^+^ cells with luciferase expression vectors under LTR control containing either intact NF-κB binding sites (NF-kB WT) or harboring mutations that prevent NF-κB interaction (MUT) [17,46,47]. Transfected cells were treated with 1 μg/mL of JALEx and luciferase activity was assessed 24 hours later. Our results showed that JALEx treatment induced a 5-fold increase in luciferase expression in cells transfected with pNF-kB WT. However, no luciferase activity was observed in cells transfected with pNF-kB MUT (Fig 4D). These results suggest that JALEx may induce HIV-1 transcription through NF-kB recruitment of its inhibitors.

### JALEx modulates surface markers expression and cytokine release in primary human CD4^+^ lymphocytes

**Fig 5:**
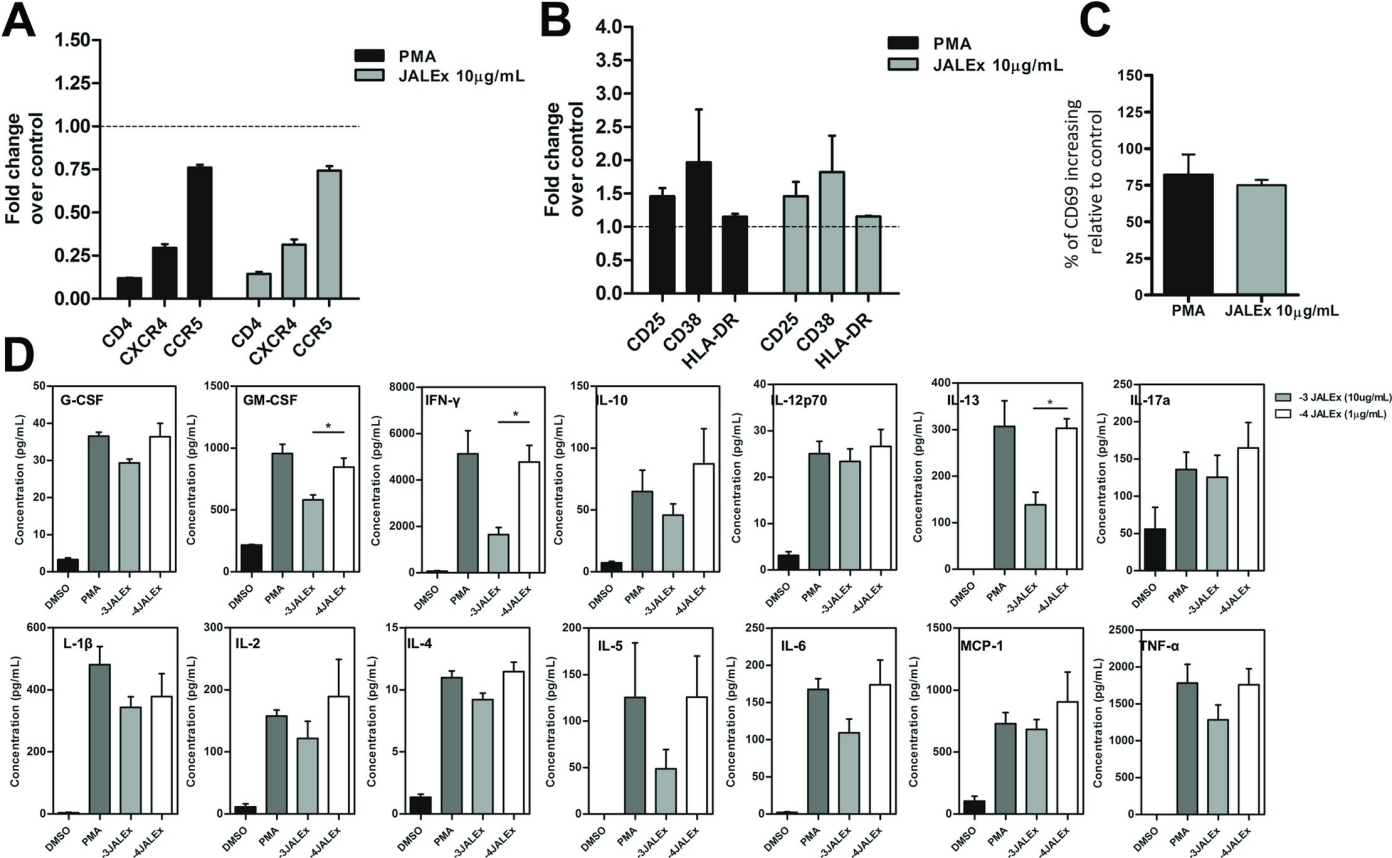
JALEx modulates the expression of cell surface receptors, activation markers and cytokine secretion in primary CD4 + T cells from HIV-1 infected individuals. CD4+ T cells were isolated from PBMCs from 4 healthy donors and treated for 24 hours with JALEx. Then, the expression of a set of surface molecules was evaluated. (A) Expression of the CD4 receptor and CCR5 and CXCR4 co-receptors in CD4 + T cells isolated after treatment with JALEx (n = 4). (B) Expression of CD25, CD38 and HLA-DR activation markers in CD4 + T cells isolated after treatment with JALEx (n = 4). (C) Increasing of the CD69 activation marker expression on CD4+ T cells isolated after treatment with JALEx extract. The black bar represents the positive control (PMA 1 μM) and the gray bar represents the treatment with 10 μg / mL of JALEx (n = 4). Data is expressed as an increasing percentage relative to controls (DMSO treated cells). (D) CD4+ T cells were treated with 1 μM PMA, 10 μg/mL (-3) or 1 μg/mL (-4) of JALEx for 24 hours. After, supernatants from the CD4+ T cells were collected and cytokine analysis were performed by Bio-plex Pro Human Cytokines 17-plex platform (n = 5, Kruskal-Wallis test and (*) indicate p<0.05). The bars in the Fig represents the SD around the mean.

To analyze whether the effects of treatment with JALEx could have an impact on primary human cells, we performed assays on CD4^+^ T cells isolated from PBMCs from 4 healthy donors. Isolated TCD4^+^ cells were treated with PMA (1 μM) or JALEx 10 μg/mL or 1 μg/mL for 24 hours. Higher doses of JALEx were used for PBMC experiments since primary cells showed greater resistance to JALEx effects compared to cell lines (S4 Fig). We evaluated the expression of HIV-1 receptor and co-receptors (CD4, CCR5 and CXCR4) and activation markers (CD25, CD38, HLA-DR and CD69) in response to JALEx treatment. We observed that JALEx treatment (1 μg/mL) downregulates the surface receptors CD4 and CXCR4 expression levels and affect CCR5 expression with less intensity (Fig 5A). The same pattern was observed for cells treated with PMA (Fig 5A). Indeed, JALEx treatment also induced the expression of surface activation markers CD25, CD38 and HLA-DR when compared to non-treated cells, and with PMA control (Fig 5B). Fig 5C shows that CD69 activation marker expression is 75 times higher relative to control cells in both treatments. Together, these results indicate that JALEx not only reactivates latent HIV-1 cells, but also could prevent new cycles of virus infection by down-modulating surface receptors used for virus entry, making this compound promising to be used as a latency-reversing agent (LRAs).

In the light of the previous results on PBMC activation, we further analyzed cytokines released directly from the supernatant of human CD4^+^ T cells under JALEx treatment. This analysis was performed using a multiplex immunoassay to quantify the production of 17 cytokines, chemokines, and growth factors. JALEx induced the production of the following cytokines relative to DMSO treated cells: IL-1β, IL-2, IL-4, IL-5, IL-6, IL-10, IL-12 (p70), IL-13, IL- 17, G-CSF, GM-CSF, MCP-1/MCAF, IFNγ and TNF-α (Fig 5D). Overall, the highest concentration of JALEx (10 μg/mL) induced the same levels of cytokines as the PMA positive control. IFN-γ and TNF-α were highly induced under JALEx treatment, 5,000 and 1,800 pg/mL, respectively (Fig 5D). This shows that JALEx activation of CD4^+^ T cells promotes the secretion of important pro-inflammatory cytokines involved in macrophage recruitment (MCP-1) and T CD8 response (INF- γ) to block HIV-1 replication. MIP-β, IL-7 e IL-8 levels were undetectable.

### JALEx increases viral load in PBMC from HIV-1 positive patients and induces Th1 and Th17 immune response

**Fig 6:**
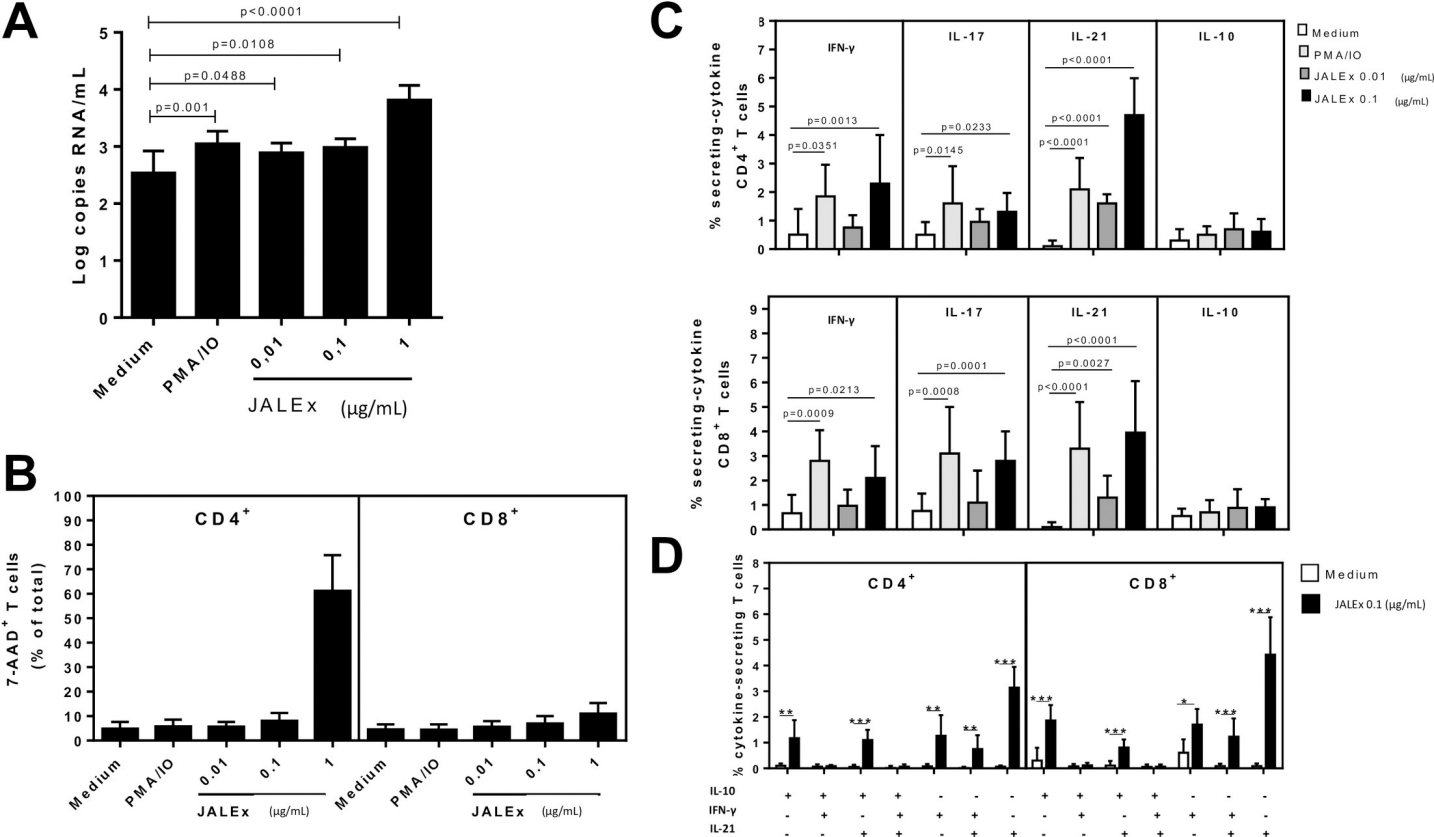
Effect of JALEx on T cell viability, HIV-1 replication in PBMC cultures and cytokine secretion in T cells from AIDS patients. Different concentrations of JALEx (0.01, 0.1 and 1 μg/mL) were added to PBMC cultures (1 x 10^6^/mL) obtained from 15 AIDS patients. 24h after, (A) the HIV-1 viral load into supernatants was determined through RT-qPCR and (B) CD4^+^ and CD8^+^ T cells viability were analyzed by FACS with 7-AAD staining. AIDS-derived PBMC cultures (1 x 10^6^/mL, n = 15) were maintained for 24h in the presence of medium alone or with different JALEx concentrations (0.01 and 0.1 μg/mL). As a positive control, specific wells were treated with PMA plus ionomycine (PMA/IO). The percentage of CD4+ and CD8+ T cells producers of IFN-γ, IL-17, IL-21 and IL-10 was determined by cytometry (C). The mean values were compared and the p-values are shown. In (D), the proportion of different (CD4+ and CD8+) T cell subsets of secreting different combinations of IL-17, IFN-γ and IL-21 in response to JALEx (0.1 μg/mL) was also evaluated by FACS. In the Fig, (*), (**), and (***) indicate p<0.05, p<0.001 and p<0.0001, respectively. The bars in the Fig represent the SD around the mean.

Progression for AIDS in HIV-1 positive patients is mainly characterized by CD4^+^ T cell depletion, specifically Th1 and Th17 cells, which impact negatively on immune protection against different opportunistic pathogens. We first analyzed the virus activation in PBMC isolated from HIV-1 positive patients under ART treatment. We found that JALEx at 1 μg/mL increases HIV-1 viral load up to 1.5 log compared with non treated PBMC cells (Fig 6A). This data was significant in all PBMC generated from the 15 HIV-1 positive patients. This increase in HIV-1 viral load was followed by an increasing of apoptotic CD4^+^ T cells (up to 82%) with no correspondence between CD8^+^ T cells (Fig 6B). In order to investigate the immune response of those patients, we evaluate cytokine production by CD4+ T and CD8+ T cells. JALEx treatment (0.1 μg/mL) increased the frequency of CD4^+^ and CD8^+^ T cells secreting IL-17, IFN-γ and IL-21 cytokines. This induction was higher compared to PMA positive controls (Fig 6C). Moreover, JALEx (0.1 μg/mL) enhanced not only the percentage of single IL-21-secreting CD4^+^ and CD8^+^ T cells, but also Th1 and Th17-like subsets positives for IL-21 (Fig 6D). In contrast, no difference was observed regarding either IFN-γ or IL-17-secreting CD4^+^ and CD8^+^ T cells from PBMC cultures of HIV-1 positive patients following JALEx treatment (0.1 μg/mL, Fig 6D). These results suggest that JALEx treatment could reactivate HIV-1 from reservoirs in patients with ART suppressive therapy and induce CD4 and CD8 positive cells to secrete protective levels of IL-21.

### JALEx reactivation effects in latently SIV-infected macaque cells

Finally, we explored the capacity of JALEx to reactivate latent virus in CD4^+^ lymphocytes from SIVmac239-infected *cynomolgus macaques*. Resting memory CD4^+^ T cells were purified from a single viremic macaque and a serial dilution replicates of these cells was prepared to seed into quantitative viral outgrowth assay (QVOA). Cells in this assay were stimulated in triplicate at each cell dilution with JALEx or another compound known to reactivate latent HIV/SIV through PKC pathway (ingenol and concanavalin A). In order to compare the ability of these compounds to reactivate latent SIV, we measured the frequency of infected cells (quantified as infectious units per million cells; IUPM) across treatments. A higher frequency of infected cells after stimulation with a given compound suggests its efficiency to reverse latency. The highest frequency of infected cells was observed in cultures treated with JALEx, although this frequency was not significantly different from those observed in other cultures (Table 1). However, JALEx was the only compound capable of reactivating SIV in all replicates tested, showing its superiority compared with others PKC agonists, such as Ingenol and Concanavalin A.

**Table 1:** QVOA results using macaques CD4+ T Lymphocytes extracted from SIV 251 infected Macaques treated with HAART therapy.

**Cells/well^a^**	**No mitogen**	**ACon A**	**Ingenol B**	**JALEx**
1.25e5	+/-/-^b^	-/-/-	+/-/+	+/+/+
6.25e4	-/-/+	-/+/+	+/+/-	+/+/+
3.1e4	+/-/+	-/-/+	-/+/+	+/+/+
Maximum likelihood (IUPM)	7.735675	5.229369	15.525127	**34.986678**
95% CI	{2.866090, 20.878852}	{1.680200, 16.275622}	{6.619641, 36.411279}	{12.619641, 66.411279}

^a^ Number of CD4+ T lymphocytes extracted from SIV 251 infected Macaques treated with HAART therapy inoculated in each cell.

^b^ Number of wells with p27 antigen positive

## Discussion

In this work we addressed the ability of an ethanolic extract obtained from *Euphorbia umbellata* latex (JALEx) to reactivate HIV/SIV latency in several *in vitro* experiments. The traditional medical uses of this Euphorbiaceae are well known in South America and this plant is vastly used for regularly consumption [24]. JALEx contains a series of bioactive phorbol esters (S1 Fig). This class of molecules is known as a PKC agonist implicated in lymphocyte activation as well as in HIV reactivation according to well-conducted *ex vivo* studies and these activities require both PKCα and PKCθ isoforms activation [18]. Some modifications at their phorbol ring can generate molecules able to induce carcinogenesis or lead to molecules that are unable to induce carcinogenesis but retain their PKC activation capacity (such as a deoxy group on ring position 4 –the 4-deoxyphorbol) [48]. The presence of this modification was identified in our JALEx during the chemical analysis and also in a previous study performed with *Euphorbia umbellata* latex [42]. Therefore, JALEx is not likely to induce carcinogenesis but it retains its the capacity to activate PKC proteins and consequently reactivate HIV/SIV latency. Many LRA act by activating PKC and several compounds such as prostatin [15], bryostatin [16], ingenol B [17], and phorbols such as PMA [18]. In this class of molecules, Prostratin and bryostratin are strong PKC agonist that are very toxic when injected intravenously. However, bryostatin was already used in a phase II study with 17 patients with progressive non-Hodgkin's lymphoma of indolent type (NHL) previously treated with chemotherapy. These patients received a median of 6 intravenous infusions of 25 μg/m^2^ bryostatin 1 given once weekly over 24h and the principal adverse effect were myalgia and phlebitis [49]. In addition, Ingenol B (a semisynthetic compound isolated from *Euphorbia tirucalli)* was previously shown as an efficient molecule to reactivate HIV-1 *ex vivo* with great potency using the PKC/NF-kB pathway inducing the cellular levels of CycT1 and CDK9 in human CD4+lymphocytes in *ex vivo* experiments [17].

In this study we used JALEx to explore its capability to reactivate HIV-1 latency taking the advantage of the common use of this plant in traditional medicine without serious side effects. We first used JALEx in J-Lat 8.4 and 10.6 cells and we could verify the potency of this extract on latent HIV transcription reactivation even in low concentrations (0.01 μg / mL). Here, we also demonstrate that JALEx acts through PKC by inhibiting J-Lat activation with three different PKC inhibitors (G6666, G6983 and Ro-31-8220). In our assays Gö6983 was the most powerful inhibitor, totally blocking HIV reactivation in both J-Lat cell lines. Go 6983 is a fast pan-PKC inhibitor of PKCα, PKCβ, PKCγ and PKCδ with its IC_50_ around a very low nM range. JALEx then could be reactivating latent HIV through the activation of different PKC isoforms. Additionally, we showed that JALEx activates the PKC pathway by promoting NF-κB internalization to the nucleus and increasing HIV-1 transcription, which apparently is dependent of the NF-κB binding site of LTR promoter. Using immunofluorescence and western blot analysis we also showed that JALEx is able to activate classical and novel PKC isoforms such as the conventional isoforms α, βI, βII and γ as well as the new ones encompassing the δ, ε, η and θ isoforms in *in vitro* experiments using JURKAT cell lines. Moreover, JALEx could induce θ isoform in high levels. The transcription factors NF-κB and AP-1 are the primary physiological targets of PKCθ, and efficient activation of these transcription factors by PKCθ requires integration of TCR and CD28 costimulatory signals. PKCθ cooperates with the protein Ser/Thr phosphatase, calcineurin, in transducing signals leading to activation of JNK, NFAT, and the *IL-2* gene. PKCθ also promotes T cell cycle progression [50].

We also evaluate the capacity of JALEx to induce cytokine production in human PBMC cells. The differences in cytokine production profiles observed for treatments with reactivating compounds is quite common on shock and kills strategies, which aims to activate cells and promote the exit of HIV from latency in T cells. This phenomenon is known as " the cytokine storm" and needs to be further studied in animal models to define a specific dose that does not induce a toxic reaction. Although JALEx induced a conjunction of pro-inflammatory cytokines we could observe an evident increase of IL-21 production by different CD4+ and CD8+T lymphocyte subsets from PBMC cultures from HIV-1 patients, which is a noble cytokine enrolled in the HIV viral load control [51–53]. For this reason, the search for new and more selective compounds that target only a few PKC isoforms or that have their mechanism of action focused on different steps of the activation pathways is necessary. Of note, leaf and latex ethanolic extract of *E*. *umbellata* is described to have low toxicity in adults rats when orally administered with acute doses (up to 2000mg/kg given orally) being well tolerated [54].

In our experiments JALEx was efficient in reactivating HIV-1 production directly in resting CD4+ T cells, in cells from HIV-1+ individuals and in primary non-human primate CD4+ T cells. The anti-HIV-1 action of JALEx could be possible by three synergistic effects: a) reactivation of HIV-1 from latency and its destruction by ART; b) blocking infection of new cells through down-modulation of CD4 on the lymphocyte surface; and c) by inducing the secretion of antiviral cytokines such as IL21. All these properties of JALEx highlight its potential for future studies *in vitro* and suggest it as a great candidate for clinical trials targeting HIV eradication.

## Supporting information

S1 Fig Phorbol esters isolated from *Euphorbia umbellata*. I = 12-*O*-Tigloyl-4-deoxyphorbol-13-isobutyrate; II = Phorbol-12,13,20-triacetate; III = 12-Deoxyphorbol-13-(12-methylpropionate); IV = 3,4,12,13-Tetraacetylphorbol-20-phenylacetate (synagrantol A); V = Deoxyphorbol-12,13-ditiglate (synagrantol B).
(TIF)

S2 Fig JALEx -mediated HIV-1 reactivation occurs via PKC activation. J-Lat cells 8.4 (left panel) and J-Lat 10.6 (right panel) were pretreated for 24 h with three different PKC inhibitors (G6666, G6363and Ro-31-8220) at the concentration of 1 μM each. The cells were then incubated with different concentrations of JALEx for an additional 24 hours and GFP expression was assessed by flow cytometry. PMA (1 μM) was used as a positive control for activation of PKC-dependent HIV-1 (n = 1).
(TIF)

S3 Fig Panel of phosphorylation of different PKC isoforms after treatment with JALEx. (A) Jurkat cells were treated with two different concentrations of JALEx (0.01 μg / mL and 0.001 μg / mL), PMA (1 μM) as positive control, at different time intervals (10, 30 minutes, 1, 6 and 24 hours). Then the cells were lysed for western blotting with phosphorylated anti-PKC antibodies (pan, δ, θ) and anti-tubulin as the loading control. (A) The intensity of the western blotting bands corresponding to the Jurkat cells in (B) that were quantified by densitometry with the aid of the Image J program. Dashed lines correspond to the band intensity of MOCK in these experiments (n = 1).
(TIF)

S4 Fig JALEx cytotoxicity assay on PBMC and HeLa cells: PBMC and HeLa cell viability measured under different concentrations of JALEx (10, 1 and 0.1 μg/mL) by CellTiter Blue vital stain after 5 days. Dashed lines indicate cell viability for DMSO treated cells that were set as 100% as a negative control for comparisons. Experiments were performed with n = 3.
(TIF)
