# Supplementary figures and images for: Reactivation of latent HIV-1 in vitro using an ethanolic extract from Euphorbia umbellata (Euphorbiaceae) latex
Source: PLoS One. 2018 Nov 27;13(11):e0207664. doi: 10.1371/journal.pone.0207664 (PMC6258530; doi:10.1371/journal.pone.0207664)

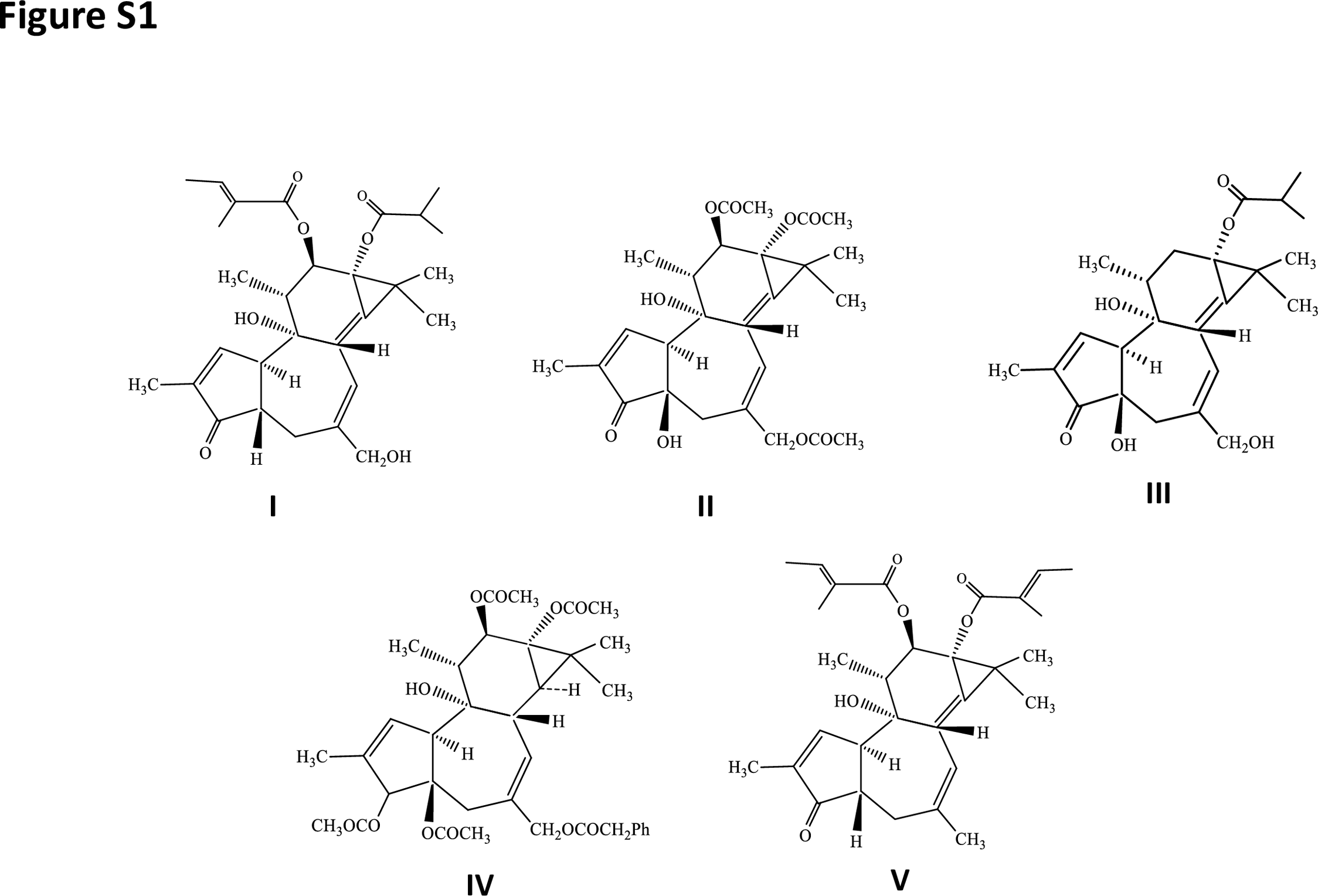

Supplement: S1 Fig — I = 12-O-Tigloyl-4-deoxyphorbol-13-isobutyrate; II = Phorbol-12,13,20-triacetate; III = 12-Deoxyphorbol-13-(12-methylpropionate); IV = 3,4,12,13-Tetraacetylphorbol-20-phenylacetate (synagrantol A); V = Deoxyphorbol-12,13-ditiglate (synagrantol B). (TIF) [file pone.0207664.s001.tif]

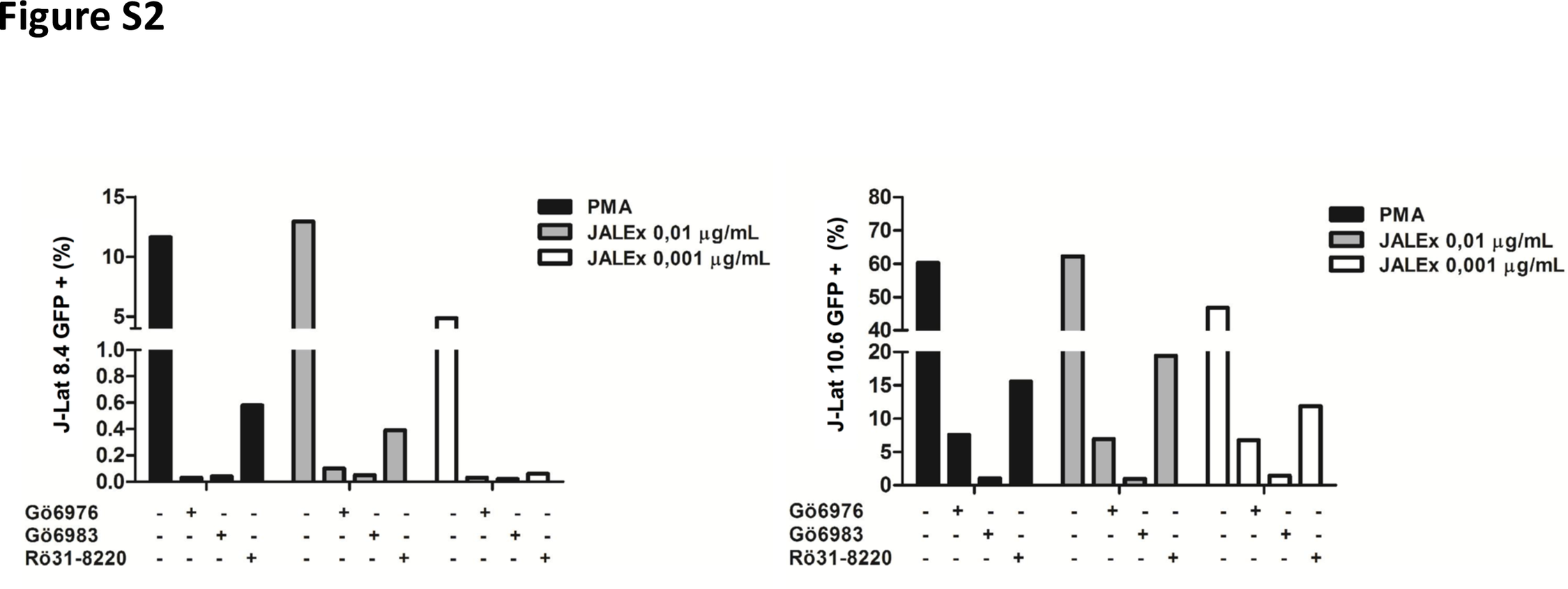

Supplement: S2 Fig — J-Lat cells 8.4 (left panel) and J-Lat 10.6 (right panel) were pretreated for 24 h with three different PKC inhibitors (G6666, G6363and Ro-31-8220) at the concentration of 1 μM each. The cells were then incubated with different concentrations of JALEx for an additional 24 hours and GFP expression was assessed by flow cytometry. PMA (1 μM) was used as a positive control for activation of PKC-dependent HIV-1 (n = 1). (TIF) [file pone.0207664.s002.tif]

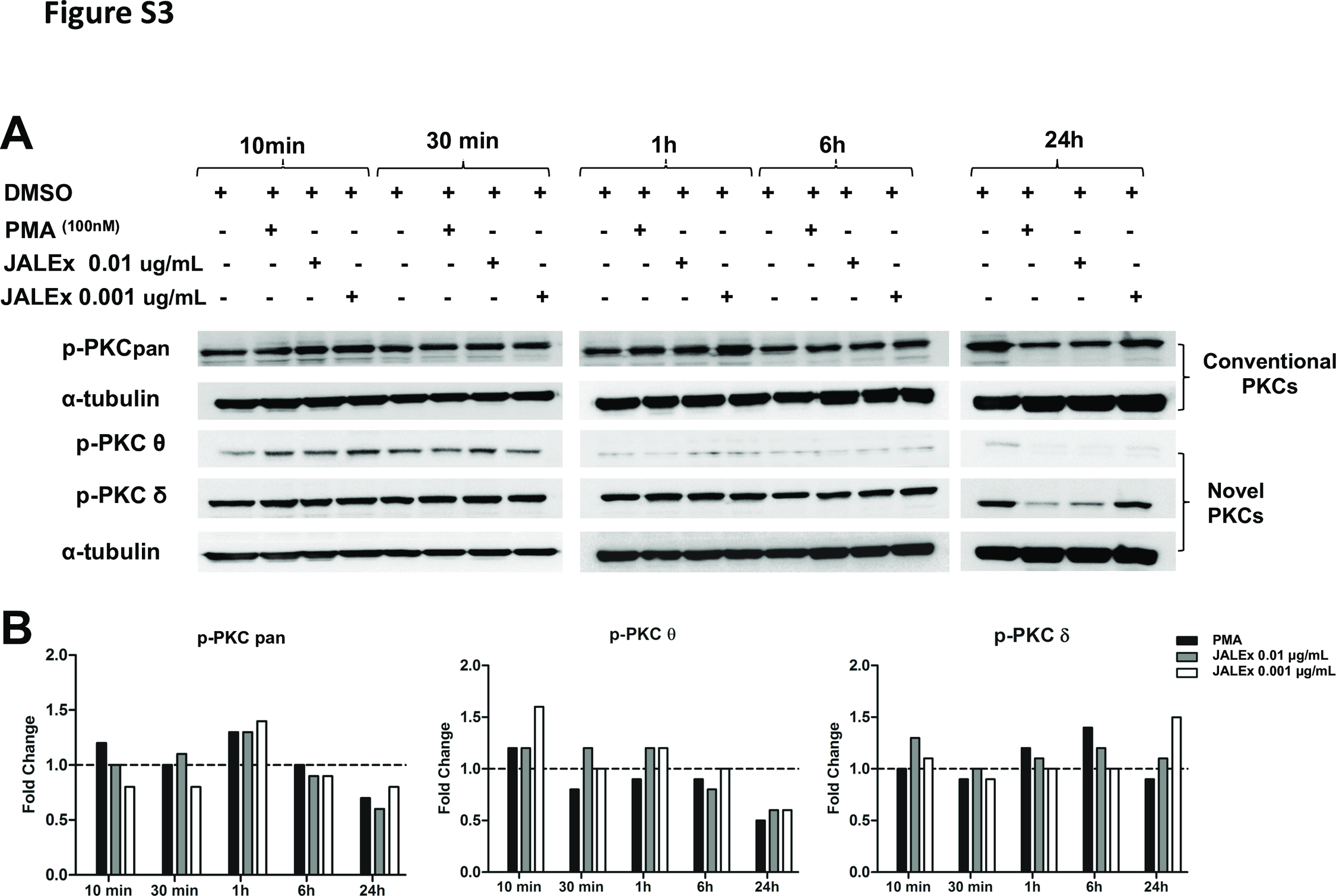

Supplement: S3 Fig — (A) Jurkat cells were treated with two different concentrations of JALEx (0.01 μg / mL and 0.001 μg / mL), PMA (1 μM) as positive control, at different time intervals (10, 30 minutes, 1, 6 and 24 hours). Then the cells were lysed for western blotting with phosphorylated anti-PKC antibodies (pan, δ, θ) and anti-tubulin as the loading control. (A) The intensity of the western blotting bands corresponding to the Jurkat cells in (B) that were quantified by densitometry with the aid of the Image J program. Dashed lines correspond to the band intensity of MOCK in these experiments (n = 1). (TIF) [file pone.0207664.s003.tif]

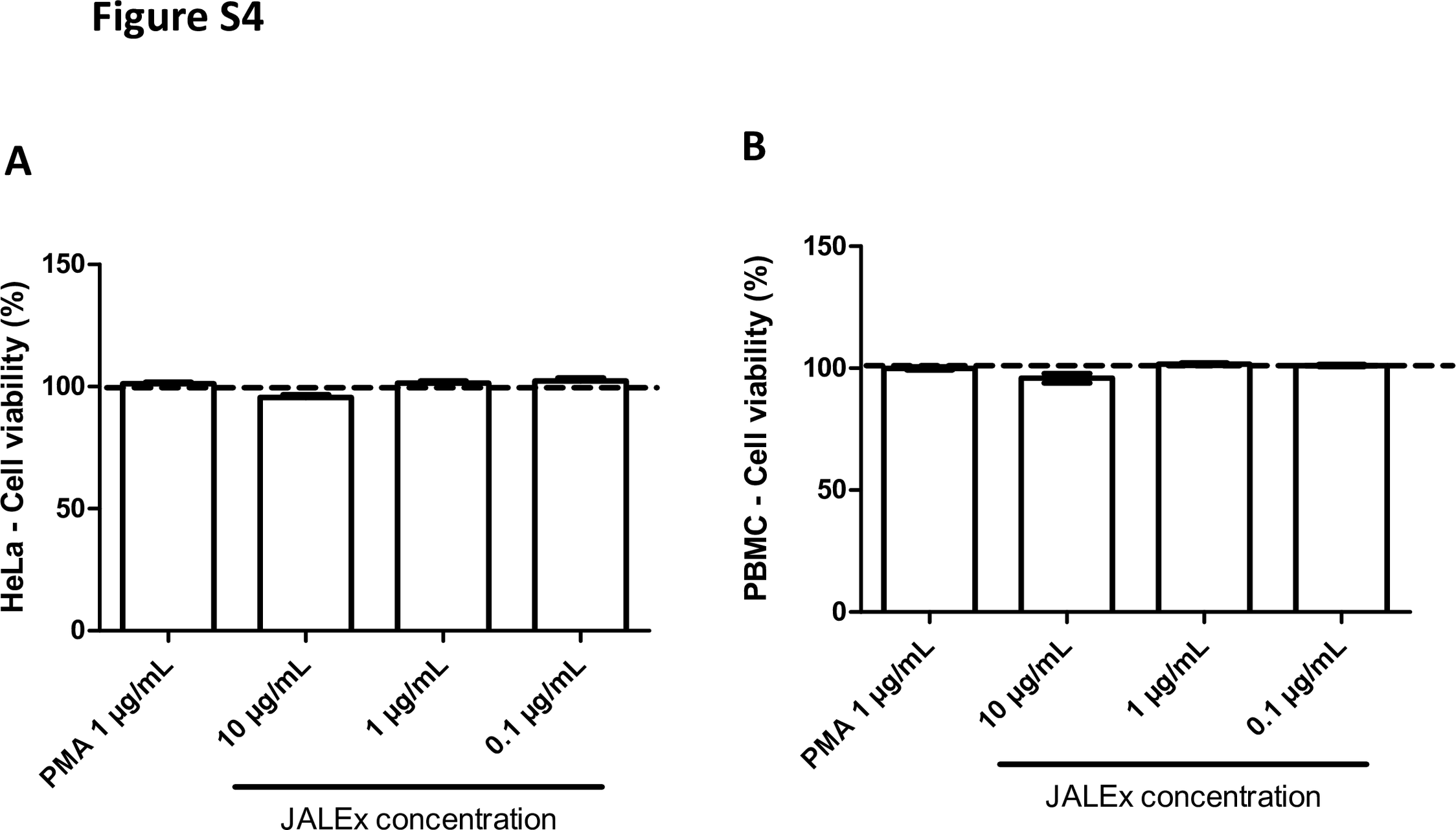

Supplement: S4 Fig — Dashed lines indicate cell viability for DMSO treated cells that were set as 100% as a negative control for comparisons. Experiments were performed with n = 3. (TIF) [file pone.0207664.s004.tif]
